# Supplementary material for: hTERT and SV40LgT Renal Cell Lines Adjust Their Transcriptional Responses After Copy Number Changes from the Parent Proximal Tubule Cells
Source: Int J Mol Sci. 2025 Apr 11;26(8):3607. doi: 10.3390/ijms26083607 (PMC12027150; doi:10.3390/ijms26083607)
Supplement: Supplementary file 1 [file ijms-26-03607-s001.zip › Figure S1 Slc22a11 Orthologs.pdf]

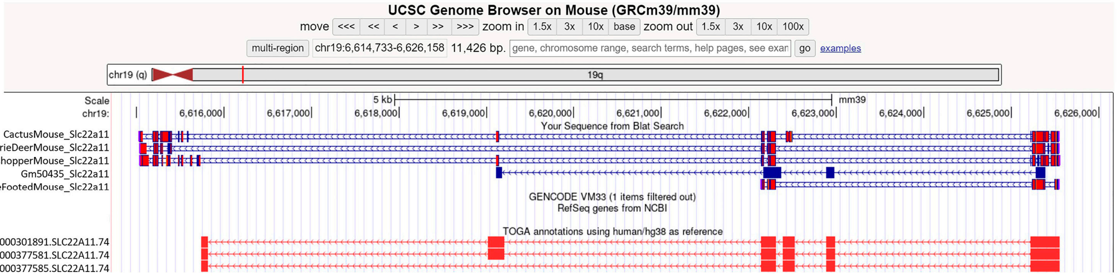

Figure S1. Slc22a11 (mOat4) transcripts alignment from related species to mouse chromosome 19. Gm50435 is the *MGI* (Mouse Genome Informatics database) gene symbol for the Havana group (Sanger Institute) predicted Slc22a11 gene on mouse Chr19: 6,619,110-6,625,392 reverse strand with ENSMUSG00000118062.2 as an Ensembl identifier. Shown above, are related mouse species genomes that contain the Slc22a11 gene and transcript orthologs that align well to Gm50435, including: the CactusMouse (Peromyscus eremicus, XM_059262142.1); Prairie deer mouse (Peromyscus maniculatus bairdii, XM_015999153.2); Grasshopper mouse (Onychomys torridus; XM_036181929.1) and White-Footed Mouse (Peromyscus leucopus; XM_037202528.1). TOGA v1.1.7 (Tool to infer Orthologs from Genome Alignments) was accessed on 9 September 2024 and is an informatic tool to predict transcripts (exons shown in red at the bottom of the figure) created by the LOEWE Centre (LOEWE-Centre for Translational Biodiversity Genomics, Frankfurt, Germany). As input, TOGA uses a gene annotation of human/hg38 as the reference species for mammals and a whole genome alignment between the reference and query genome. TOGA predicted three splice variants as Slc22a11 protein coding transcripts with Ensembl identifiers shown at the left of each transcript variant.
